# Training-Based Methods for Comparison of Object Detection Methods for Visual Object Tracking

**DOI:** 10.3390/s18113994

**Published:** 2018-11-16

**Authors:** Ahmad Delforouzi, Bhargav Pamarthi, Marcin Grzegorzek

**Affiliations:** Research Group for Pattern Recognition, University of Siegen, Hölderlinstr. 3, 57076 Siegen, Germany; p.bhargav379@gmail.com (B.P.); marcin.grzegorzek@uni-siegen.de (M.G.)

**Keywords:** object tracking, deep learning, object detection, online training, Kalman filter

## Abstract

Object tracking in challenging videos is a hot topic in machine vision. Recently, novel training-based detectors, especially using the powerful deep learning schemes, have been proposed to detect objects in still images. However, there is still a semantic gap between the object detectors and higher level applications like object tracking in videos. This paper presents a comparative study of outstanding learning-based object detectors such as ACF, Region-Based Convolutional Neural Network (RCNN), FastRCNN, FasterRCNN and You Only Look Once (YOLO) for object tracking. We use an online and offline training method for tracking. The online tracker trains the detectors with a generated synthetic set of images from the object of interest in the first frame. Then, the detectors detect the objects of interest in the next frames. The detector is updated online by using the detected objects from the last frames of the video. The offline tracker uses the detector for object detection in still images and then a tracker based on Kalman filter associates the objects among video frames. Our research is performed on a TLD dataset which contains challenging situations for tracking. Source codes and implementation details for the trackers are published to make both the reproduction of the results reported in this paper and the re-use and further development of the trackers for other researchers. The results demonstrate that ACF and YOLO trackers show more stability than the other trackers.

## 1. Introduction

**Figure 1:**
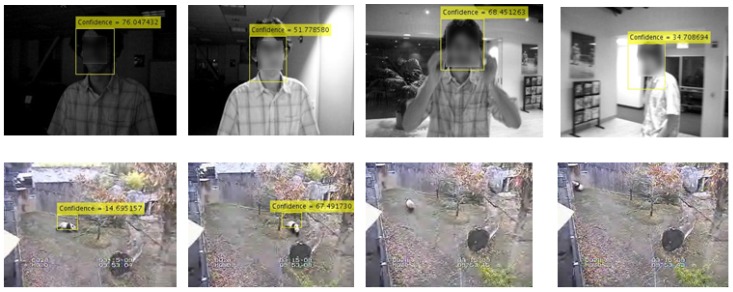
Challenges in two videos: First line (David) and second line (Panda) show illumination change, out-of-plane rotation, appearance change and background clutter.

With knowing the location of a desired object in the first frame, the task of tracking of this object becomes a fascinating topic for video processing from both scientific and industrial viewpoints. This task becomes scientifically more interesting when there is complexity involved in video sequences. This complexity can include a moving camera, uncertainty of the object, background clutter, small size and low resolution of the object, size variation, appearance changes, occlusion, articular objects, illumination change and out-of-plane rotation. Some of the challenges are shown in Figure 1.

Recently many methods have been developed to overcome the challenging object tracking problem in videos. Some trackers focus on human tracking with various techniques such as common energy minimizing [1,2] and data association [3], and some other trackers focus on object tracking using various methods like point tracking [4] and feature descriptors [5]. Multiple cues such as color, shape, and position are selected as human tracking features. In the case of fixed camera scenarios, the background is still. Thus, background detection and subtraction allow foreground object detection. In other words, looking for only moving objects in a limited area allows finding objects of interest. There are many other methods for human detection and tracking in literature [1,2,3,6,7,8,9,10]. These methods often use very simple features to detect humans. The human body is usually described by some simple shapes such as a circular for the top part and a cylindrical shape for other body parts. Thus, a very commonly used method is modeling the human appearance to 2d shapes [7] or 3d shapes [8]. Some other methods use offline-trained objects for human tracking problem [3]. In [9], authors use Edgelet-base detectors for human tracking.

Each tracker has its own strength and weakness. For instance, the tracking problem can be formulated as a correlation between successive frames [11,12,13,14]. It means the location of the object in the current frame is a region or candidate with a maximum correlation to the object of interest in last frame. Liu et al. [11] addressed illumination variation by using a correlation filter-based tracker. Fog computing platform was used to speed up tracking. The correlation tracker [12] is robust to some challenges. However, since the model that the KCF trackers learn depends strongly on the spatial layout of the tracked object, they are fragile to deformation, occlusion and camera shaking. In a method presented in [15], authors compensate this drawback by combining the correlation based system to color information. Online training method [16] can handle occlusion well but it is fragile to the out-of-plane rotation.

Object tracking can be formulated in terms of object detection. For example, SURF features [17], which were originally proposed for object detection, can be used for object tracking. In [5], authors employ and compares SURF and SIFT features for object tracking and shows that SURF features have better results. SURF-based object tracking methods were presented in [18,19], but they are unable to handle occlusion and fragile to background clutter. SURF-tracker is usually combined with other methods to improve the performance. In [20], a combination of the SURF and camshift methods was used for object tracking in an indoor environment. In a method presented in [21], authors combine mean-shift, SURF and a two-stage matching for tracking. In [22], a dynamic object model and the surf features are used for human tracking.

Chen et al. [23] introduces a learned linear regression models through single convolutional layer with the gradient descent technique. It outperforms most correlation filter-based trackers. In a method presented in [24], combines 6 different trackers in a winner-take-all framework to improve the strength of the overall tracker against various challenges compared to the individual trackers. The selection method of the trackers is based on a performance prediction model. In [25], authors proposed two types of tracklet interactions for the multi-object tracking. Close interaction detects near objects and distant interaction accounts for the higher order motion and appearance.

Ding et al. [25] modeled the data association of the tracking problem as a flexible quadratic binary energy minimization, and solved it with the efficient QPBO technique. In [26], authors proposed a long-term motion tracker for the intelligent vehicles. A set of independent classifiers were trained sequentially on different small datasets. Each classifier was used to solve certain different sub-problems occurred in a non-stationary environment. Guan et al. [27] proposed a modular tracker for event-triggered tracking in the presence of model drift and occlusion. It is composed of a short-term tracker, occlusion and drift identification, target re-detection, short-term tracker updating and online discriminative learning of detector. In a method presented in [28], authors proposed a semantics-aware object tracking method, which introduced semantics into the tracking procedure to improve the robustness of tracking. In [29] authors use FasterRCNN for object detection and a sequential color particle filtering for tracking. Kim et al. [30] combines long short term memory (LSTM), a residual framework and another LSTM to build an attention network for object tracking. It uses LSTM for temporal learning of object tracking. A learning-based tracker using a Bayesian estimation framework is proposed in [31]. It uses different blurring kernels to increase the robustness of the tracker against blurring.

Yun et al. [32] use an offline convolutional deep neural network (ADNet) for object detection and an action-driven method for temporal tracking. In a method presented in [33], authors proposed a learning-based tracking method which uses deep appearance to learn a discriminative appearance model from large training datasets for a reliable association between tracklets and detections.

In a method presented in [34], authors propose an object tracking method based on transfer learning. It trains an autoencoder by using auxiliary natural images as feature extractor in offline and then uses an additional classification layer in online. In [35], authors use transfer learning for object tracking. Some layers in an offline-trained CNN are transferred to an online classifier with an updating binary classifier layer. This classifier produces some candidates around the previous target which are further evaluated to output the target. In a method presented in [36], authors use recurrent neural networks for the task of tracking objects in 2D laser data in robotics applications. In [37], authors use oblique random forests for object tracking. It uses HOG features and deep neural network-based models as the features. An incremental update steps to update the tracker. In a method presented in [38], authors propose a kernel cross-correlator to improve the robustness of linear cross-correlator-based trackers. It can handle affine transformations.

Recently, there is a drastic progress in the area of object detection by using learning-based techniques like deep learning. However, this progress was not extended to trackers. In this paper, five famous training-based object detectors i.e., ACF [39], RCNN [40], FastRCNN [41], FasterRCNN [42] and you only look once (YOLO) [43] are considered for object tracking and a comparative study among the detectors is done in this context. Two methods for offline tracking (training before tracking) and online tracking (training while tracking) are used. The former uses a pre-trained model for object detection in the space dimension (i.e., still images) and another offline trained classifier for the association of the objects in the time dimension. The latter is a short-term tracker with an online training procedure which updates the detector over the time. In other words, the offline tracker divides the tracking task into two separate tasks of detection of objects in frames and finding the object of interest among the objects of each frame.

The object detection term refers to find an object in an image and object tracking means to follow an object of interest within a group of frames in a video. In this paper, the trackers follow the object of interest using a tracking-by-detection method. The tracking-by-detection means the same object is detected in successive frames of the video.

The object detector performs the first part and the second part is a time series analyzer for tracking. The online tracker trains a detector with the positive and negative data generated from the first frame and then the detector is applied to the next frames of a certain part of the video, the detector is re-trained with the recently detected objects within the video part.

To the best knowledge of the authors, the five mentioned detectors have not previously been compared in online and offline trackers. The online tracker is far different from two-step trackers [29,30,32,33] which first detect objects in images and then associate the detected objects using another classifier. In contrast with [34,35], the online tracker does not have any offline phase. It also does not transfer layers from another network.

The rest of the paper is organized as follows: Section 2 describes and compares the exploited training based detectors. Then, the object tracking methods are explained in Section 3. The experimental results, evaluations and comparisons and discussions are shown in Section 4. Section 5 concludes the paper.

## 2. Training Based Object Detection

This section explains the new object detectors i.e., ACF [39], RCNN [40], FastRCNN [41], FasterRCNN [42] and YOLO [43] which are used for object tracking in this paper. These detectors were selected because they are famous object detectors and easy to use.

### 2.1. Aggregate Channel Features

For an ACF (Aggregate Channel Features) detector, a channel refers to a certain component that defines pixel values in image [39]. ACF then generates many features by using obtained channels. These features are called channel features and can be mainly categorized into two types: first- and high-order channels [39]. ACF extracts First-order channel features from a single channel by summing pixels and higher-order channel features by combining two or more first-order channel features. ACF then uses decision trees for classification.

### 2.2. Region-Based Convolutional Neural Network

Region-Based Convolutional Neural Network (RCNN) is an object detector based on Convolutional Neural Network (CNN). CNN performs convolution products on small patches of the input map of the layer. Thus, extracting features are carrying information about local patterns [44]. A typical Convolutional Neural Network (CNN) is composed of two main layers of the convolutional and fully connected layers [45]. First, RCNN computes the region proposal using selective search [46]. Then, it forwards the proposals to a trained Convolutional Neural Network.

The region proposals with greater than 0.5 IoU (Intersection over Union) overlap with a ground truth (defined by a user) are classified as positive and the rest of the proposals are classified as negative. The RCNN has 3 convolutional layers and 2 fully connected layers. RCNN is slow because it performs a CNN for each proposal, without computation sharing.

### 2.3. Fast Region Based CNN

FastRCNN made improvements to RCNN to increase the process speed. It shares computation of the convolution layers among different proposals. Since the convolutional layer does not change the spatial relationship between the adjacent pixels, it projects coordinates in the raw image to corresponding neurons in the convolutional layer. Therefore, the whole image can be computed through the convolutional layer once and the processing time is saved [41]. FastRCNN is up to ten times faster than RCNN but it is not real-time.

### 2.4. Faster Region-Based CNN

FasterRCNN [42] brings object detection toward a real-time application. It uses a region proposal network (RPN) after the last convolutional layer. RPN takes an image feature map as input and outputs a set of rectangular object proposals. The RPN network detects whether the current region, which is generated from a sliding window and different anchors (for each location, some proposals with different aspect ration are parametrized relative to their reference boxes are called anchors), is the object of interest. FasterRCNN is up to ten times faster than FastRCNN and it is real-time.

### 2.5. YOLO

YOLO is state-of-the-art in object detection. It uses deep networks and a hierarchical structure for object labeling and classification real-time [43]. YOLO stands for you only look once, i.e., it just looks at the image once and processes simultaneously the whole image. Instead of a large softmax in Imagenet [47], YOLO uses several softmaxes as a hierarchical tree, each softmax decides for a similar group of objects. In this way, YOLO classifies objects more accurately than Imagenet. It is faster than VGG-16 [48] because it uses few floating point operations. It applies a single convolutional neural network to the full image. This detector divides the image into regions and predicts bounding boxes and a probability for each region. These bounding boxes are weighted by the predicted probabilities. Like FasterRCNN, it adjusts priors on bounding boxes instead of predicting the width and height outright. However, it still predicts the *x* and *y* coordinates directly. YOLO is faster and more precise than FasterRCNN.

## 3. Object Trackers

### 3.1. Online Tracker

**Figure 2:**
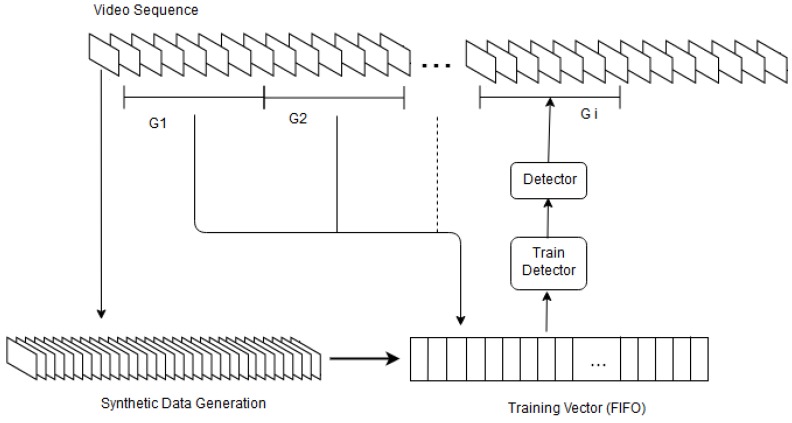
Block diagram of the online tracker. In the first frame, from this selected object, synthetic data is generated. Then a detector (i.e., RCNN, FastRCNN, FasterRCNN or ACF) is trained using the generated data and applied to the first segment of frames $G_{1}$ to detect the objects of interest in them. The detected objects and the synthetic data added to the training vector $T_{v}$ which is then used to update the detector. This process is continued until the end of the video.

The steps of the online object tracking method are shown in Figure 2. In the first frame, a user selects an object of interest. From this selected object, the online tracker generates synthetic data. This procedure is described in Section 3.1.1. The tracker trains a detector using the synthetic data. It then segments the input video frames into unequal-length groups of frames $\left\{G_{1},G_{2},\ldots ,G_{n}\right\}$. Each group is composed of several frames i.e., $G_{1}=\left\{f_{2},\ldots ,f_{m1}\right\},G_{2}=\left\{f_{m1+1},f_{m1+2},\ldots ,f_{m2}\right\},\ldots ,G_{n}=\left\{f_{m(n-1)+1},f_{m(n-1)+2},\ldots ,f_{mn}\right\}$.The trained detector tracks the first group i.e., $G_{1}$ using the detection of objects of interest within $G_{1}$. This process is explained in Section 3.1.1. The objects of interest are a pedestrian, a Panda, a car and so on. At the end of the first iteration, the online tracker copies the detected objects within $G_{1}$ as well as the synthetic data to a training vector $T_{v}$ (see Figure 2). The tracker uses the training vector $T_{v}$ to train the detector for the second time and then applies it on the second segment $G_{2}$ and then concatenates the detected objects within $G_{2}$ to the training vector $T_{v}$. The tracker updates steadily the training vector prior to each training iteration until the last frame of the video. It updates the detector by using a first input first output(FIFO) procedure. Thus, the tracker follows recent appearances of the object.

In the section of experiments, we investigate the length of the training vector in terms of accuracy. Let $I_{t}^{x,y}$ denote the pixels of a single frame *t*, then the following equation expresses the online tracker output for this frame (i.e., $T_{t}^{n}$):

(1) $$T_{t}^{n}=D\left(I_{t}^{x,y},W^{n}\left(t\right)\right)$$

In this equation, D(.) is the detector response which is a function of the image pixels and a trained network with the weights of $W^{n}\left(t\right)$. While updating the detector *D* in a certain interval Δt (shown in Figure 2 using $G_{1},G_{2}$, ...), the weights $W^{n}\left(t\right)$ are updated by using the generated samples as well as the detected objects from the first frame to the last frame (t−1). Equation (2) explains it. The tracker calculates initial weights for detection of the object of interest in the second frame using *N* synthetic positive and negative data from the first frame (i.e., $W^{n}\left(1\right)=F\left(I_{1}^{1},I_{1}^{2},\ldots ,I_{1}^{N}\right)$).

(2) $$W^{n}\left(t\right)=F\left(I_{1}^{1},I_{1}^{2},\ldots ,I_{1}^{N},I_{2}^{d},I_{3}^{d},\ldots ,I_{t-1}^{d}\right)$$

where $I_{i}^{d}$ is the object of interest of for frame *i*, e.g., $I_{t-1}^{d}$ is the object of interest for the last frame. By using the online tracker and the four detectors of ACF, RCNN, FastRCNN and FasterRCNN, we use four online trackers and compare them in Section 4.

#### 3.1.1. Synthetic Data Generation

To have an effective tracker, the detectors must be trained using enough number of training data. The variety of data is very important factor in the training process. To have such a diversity, the following process is proposed:

1. Rotated copies of the object of interest using the rotating angles {−10, −9.9, −9.8, ..., 0, ..., 9.9, 10}.
2. Additive salt and pepper noise, with noise densities {0.001, 0.002, ..., 0.008 }.
3. The enhanced version of the rotated images in item 1 using contrast adjustment .
4. The enhanced version of the rotated images in item 1 using histogram equalization.
5. Increasing and decreasing of image brightness with the brightness factor {0.81, 0.82, 0.83, ..., 1.29, 1.3}.
6. Resized version of the object of interest using the resizing factor {0.555, 0.560, 0.565, ..., 1.55}.

The tracker combines the above items to generate diverse data. We use different total number of the synthetic data in our experiments (i.e., 500, 800, 1000, 2000, 4000 and 10,000) to investigate its effect on tracking accuracy. The number and types of the synthetic data are given in Table 1.

The above parameters are selected in an optimized way to preserve both tracker accuracy and speed. Initially, a small training set was chosen and poor results were obtained. Then, the above parameters were gradually optimized to maximize the tracking accuracy and speed. Therefore, they are independent on type of objects and videos. The detailed information regarding the parameter optimization is given in Section 4.2.

**Figure 3:**
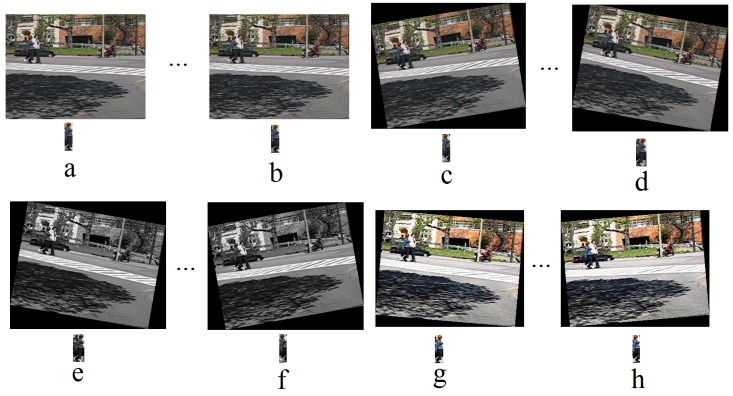
Synthetic data for the training of the detectors with their objects of interest, the first frame of “Pedestrain1” (**a**), this frame with additive salt and pepper noise, with noise density 0.008 (**b**), rotated version of (**a**) with −10 and 9 degree (**c**,**d**), rotated version of the enhanced image (**a**) using histogram equalization (**e**,**f**) and using contrast adjustment (**g**,**h**).

Figure 3 shows some examples of the synthetic data for “Pedestrain1”. Figure 3a shows the first frame and the selected object (object of interest), this frame is added by salt and pepper noise, with noise density 0.008 (Figure 3b) and rotated with −10 and 9 degree (shown in Figure 3c,d), enhanced using histogram equalization and then rotated with 9 and −10 degree (shown in Figure 3e,f) and enhanced using contrast adjustment and then rotated with 5 and −5 degree (shown in Figure 3g,h). The object of interest for the selected frames is shown below of each image.

#### 3.1.2. Tracker Updating

The detected objects within frames of the first group $G_{1}$ (i.e., {$I_{2}^{d},I_{3}^{d},\ldots ,I_{m1}^{d}\}$) are concatenated to the training vector $T_{v}=\left\{syntheticdata,I_{2}^{d},I_{3}^{d},\ldots ,I_{m1}^{d}\right\}$. By using the updated training vector, the tracker trains the detector. The updated detector is then used for tracking of frames (detection of the object of interest) in the second group $G_{2}$. The detected objects within frames of $G_{2}$ are again concatenated to the training vector $T_{v}$ and the updated $T_{v}$ is used for tracking in the third group $G_{3}$. The length of the groups $\left\{G_{1},G_{2},\ldots ,G_{n}\right\}$ is obtained from Equation (3). During the training, when the $T_{v}$ gets full, the data at the beginning of the training vector $T_{v}$ is replaced by the data from the current image (FIFO vector). This process continues until end of the video. The length of $T_{v}$ is investigated in terms of the tracker accuracy in Section 4.

The length of each group $U_{s}\left(k\right)$ is calculated from the following Equation:

(3) $$U_{s}\left(k\right)=\left\{\begin{array}{cc} S_{1} & :k<T_{1}\\ S_{2} & :T_{2}>k\geq T_{1}\\ S_{3} & :T_{3}>k\geq T_{2}\\ S_{4} & :T_{4}>k\geq T_{3}\\ S_{5} & :T_{5}>k\geq T_{4}\\ S_{6} & :T_{6}>k\geq T_{5}\\ NoUpdate & :k\geq T_{6} \end{array}\right.$$

where $S_{1},\ldots ,S_{6}$ are the length of the group of frames, *k* is the frame index, and $T_{1},\ldots ,T_{6}$ are the length thresholds according to Table 2. In our experiments for ACF we choose 2 sets of parameter SET1 and SET1 (shown in Table 2) and call them ACF1 and ACF2 respectively. We choose SET2 for RCNN, FastRCNN and FasterRCNN.

### 3.2. Offline Tracker

**Figure 4:**
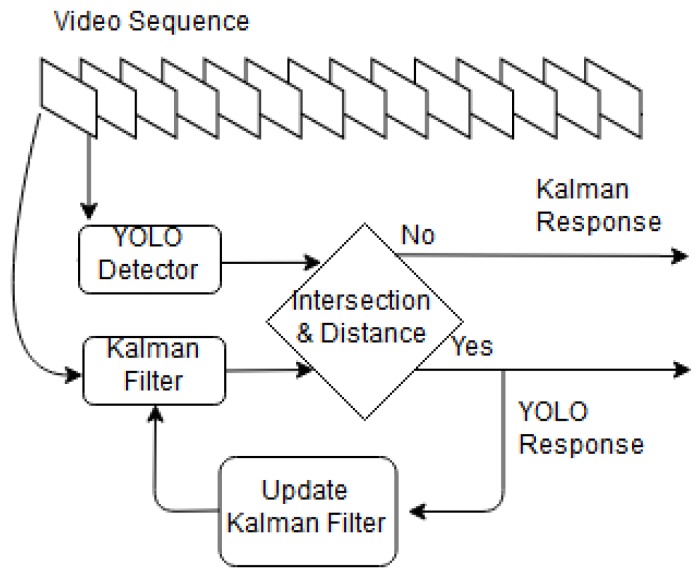
Block diagram of the offline tracker. All frames are fed to YOLO and Kalman filter. The offline tracker outputs the YOLO response which has maximum IoU with the estimated pose of Kalman filter. The YOLO response also updates the Kalman filter.

The offline tracker steps are shown in Figure 4. Thw video frames are fed to YOLO and Kalman filter [49]. The offline tracker output (T(.)) is the YOLO response with maximum IoU of the estimated pose by Kalman filter. If there is not intersection between the two responses, the offline tracker selects the YOLO response with lowest Euclidean distance to the Kalman filter response. The Kalman filter response is a point with the maximum probability of object presence in frame. A bounding box with the same size of the object in the last frame (k−1) is drawn around the point to show the object region. The offline tracker $T_{t}^{f}$ can be expressed using the following equation:

(4) $$T_{t}^{f}=T\left(D\left(I_{t}^{x,y},W^{d}\right),K\left(I_{t}^{x,y}\right)\right)$$

Unlike the online tracker (1), the weight of offline detector $W^{d}$ is not updated during tracking.

For more detailed information about the Kalman filter we refer to [50,51]. We use object position $p(p_{x},p_{y})$ (center of mass) and its velocity $v(v_{x},v_{y})$ and acceleration $a(a_{x},a_{y})$ (see the following Equations) for Kalman filter.

(5) $$v_{k}=p_{k}-p_{k-1}$$

(6) $$a_{k}=v_{k}-v_{k-1}$$

If YOLO does not detect any object in a frame or if the Euclidean distance between the Kalman filter response and the nearest YOLO response is more than a predefined threshold (100 pixels), then the object is considered to be occluded with other objects or left the scene. YOLO threshold value is set to 0.15 to minimize the probable object missing. Lower and higher values lead to high false alarm rate and object missing respectively. Since YOLO has a strong pre-trained model for object detection (available in [52]) and due to its slow training, we use YOLO in the offline tracker. Our initial experiments showed that for our dataset the detection rate of YOLO is high, but its classification is not precise. Therefore, we ignore the output labels of YOLO.

**Figure 5:**
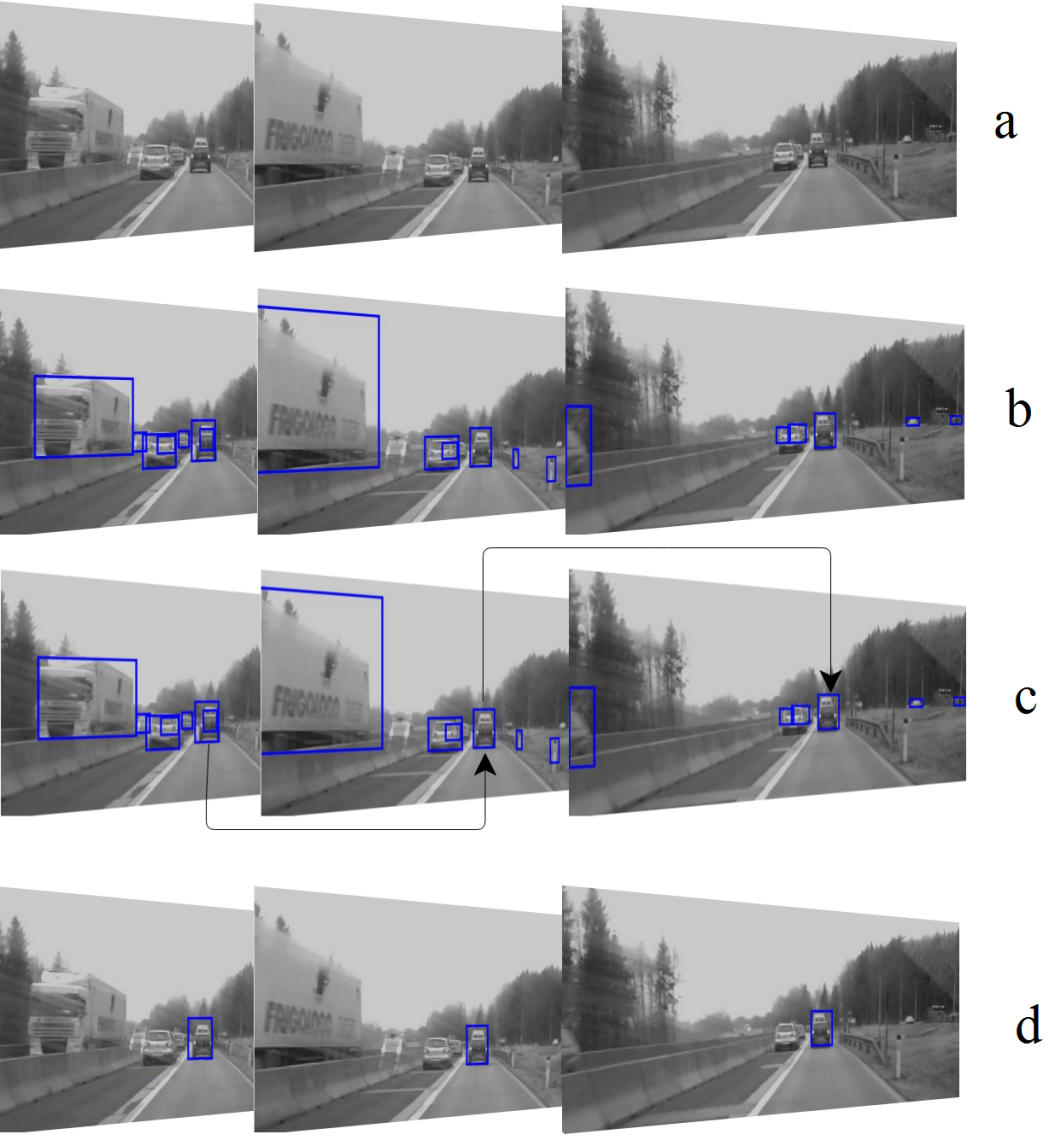
Intermediate results of the offline tracker: (**a**) Three frames of Volkswagen (**b**) The results of YOLO detector on the frames (**c**) Applying of the Kalman filter on the detected objects of (**b**,**d**) The final results of the offline tracker.

Intermediate results of the offline tracker are shown in Figure 5. First, YOLO detects all the objects in the scene. Then, the Kalman filter selects the object of interest within each frame and associates them. In this case, two connected objects (i.e., VW and the white van) are detected as a single object. This problem originates from YOLO-detector misdetection. However, since the common area between the detected object and ground truth is more than 50 percent of the ground truth area, it is seen as a true detection.

## 4. Results

### 4.1. Dataset and Parameters

**Figure 6:**
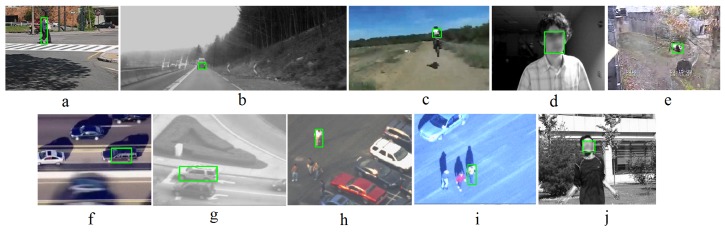
The snapshots of videos in our experiments. The figures from (**a**–**j**) show David, Jump, Pedestrain1, Pedestrain2, Pedestrain3, Car, Motocross, VW, Carchase and Panda respectively. The ground truth is shown on each image with one green rectangle.

To validate the trackers, one set of 10 videos from TLD [16] and VOT [53] datasets including more than 26,800 frames and various objects of interest are selected. The datasets contain various types of tracking challenges like Moving camera, long videos, Object partial and full occlusion and appearance, illumination, scale change and similar objects [16,53]. Some videos in TLD dataset are also available in other datasets like carchase and pedestrain1 in VOT2018. Some videos are similar to others in the VOT2018. For instance, “Volkswagen” is similar to “LiverRun”, “Car1” and “Yamaha” and “Traffic” are similar to “Motocross” and so on. They include various desirable objects for tracking like a car, a motorcycle, a car, a pedestrian, the human face, the human body and a panda as shown in Figure 6. Green rectangles in Figure 6 show the objects of interest. Among the objects of interest, car is rigid and the other objects are articular. The datasets include short and long videos. To evaluate the trackers. The sequences were manually annotated as ground truth [16]. The plane rotation more than 50% was annotated as “not visible” [16].

The parameters concerning the online trackers are set according to Table 3.

These parameters were selected using initial experiments to preserve both accuracy and speed of the trackers. Since the datasets contain various type of objects with different sizes and shapes, in different scenes, backgrounds, illumination conditions and different degrees of occlusion, the generality of the selected parameters is guaranteed.

For the synthetic data as mentioned in Section 3.1.1, the first frame of the video is exposed to a salt and pepper noise, rotation, intensity adjustment, histogram equalization, brightness change, resizing and contrast enhancement. The total number and the types of the synthetic data are shown in Table 1.

### 4.2. Accuracy Results

The experiments in this section use the following evaluation procedure. The trackers are initialized in the first frame of a video sequence and track the object of interest (shown in Figure 6) up to the end. The produced trajectory is then compared to ground truth using the recall*R*, the precision*P* and the F-measure*F*. The F-measure is calculated using F=2PR/(P+R). For each frame with a detected object, the object is considered to be correctly detected, If the common area between the detected object and the ground truth is more than 50 percent [16]. The number of selected videos is equal to videos used in other outstanding tracking methods like [16].

The detailed results of the trackers on the datasets in terms of Recall, Precision and F-measure are shown in Table 4.

ACF2 shows better performance than ACF1 for most of the videos. It shows that the minimum segment size of frames groups should be set to 20 (shown in Table 2 parameter SET2 has better results than set1.) and the smaller change in the step size (i.e., 20, 30, 40) leads to better results. In these beginning of the video, the online tracker is not well trained. Therefore, it should be updated in shorter intervals. The online tracker gradually adapts itself to these object and the scene and therefore longer updates are suitable. We trained the online RCNN1 tracker and the online RCNN2 tracker with SET1 and SET2 (Table 2) respectively. RCNN2 is better than RCNN1 for most of the videos. This experiment shows the preference of SET2 to SET1. SET2 was chosen for the other online trackers i.e., FastRCNN and FasterRCNN. FastRCNN and FasterRCNN show worse results than RCNN and ACF for most of the videos. For the 4 cases of Carchase, Jump, Pedestrain1 and Pedestrain2 fasterRCNN shows better result than RCNN and FastRCNN, but for 6 other videos RCNN is better. It shows that FasterRCNN shows better performance in the presence of similar objects. Among the online trackers, ACF2 has the best overall robustness but the YOLO tracker is even more stable than ACF2.

**Figure 7:**
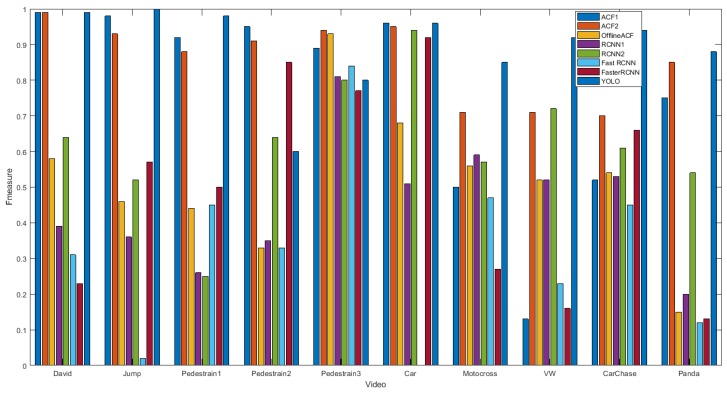
Tracker comparison in terms of F-measure.

Figure 7 compares the F-measure of the trackers.

In another experiment, we changed the training vector length $T_{v}$, the number of the training iterations and the total number of the synthetic data. We tried different lengths of the synthetic data i.e., 500, 1000, 2000, 4000 and 10,000 with different training iterations of 3 and 10. Then, we calculated the online-trackers F-measure.

**Figure 8:**
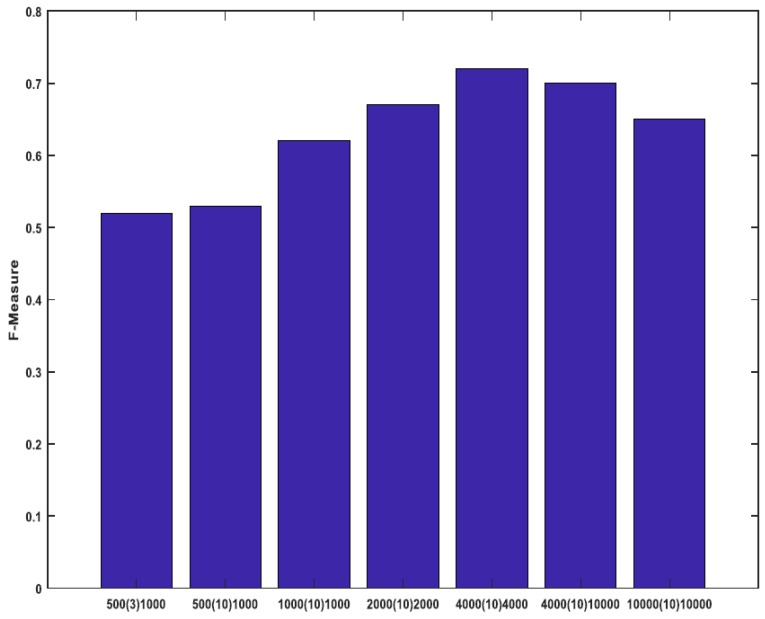
The trackers performance versus the synthetic data length (left number), the training iteration number (middle number) and the training vector $T_{v}$ length (right number).

The trackers stability using average F-measure is shown in Figure 8. When we increase the synthetic data length from 500 to 4000, the performance increases. However, for the lengths bigger than 4000, it decreases. Thus, the optimum number of synthetic data length is 4000. With a training iteration increasing from 3 to 10, the trackers stability increases. With further increasing, the tracker speed decreases but the accuracy improvement is very little. The training vector length $T_{v}$ is increased from 1000 to 10,000. In this case, when $T_{v}$ exceeds 4000 (other parameters do not change) the accuracy falls down. Thus, the optimum $T_{v}$ length is 4000.

**Figure 9:**
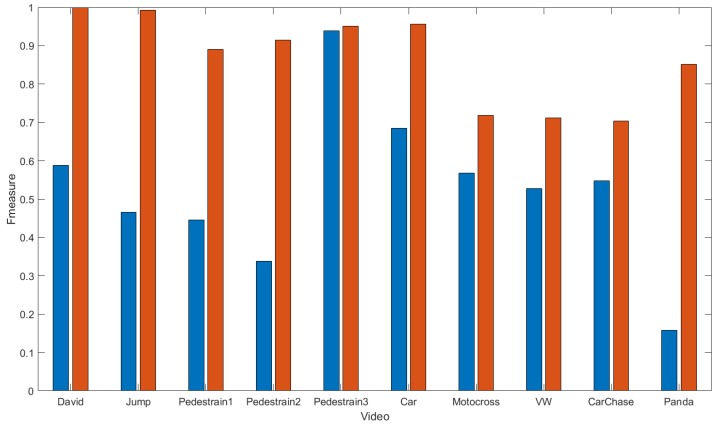
In the ACF tracker, the updating process was removed. The results of the online tracker and the ablative study are compared.

In an ablative study on the online ACF tracker, we removed the updating/training process. The results of the study are shown and compared in Figure 9. According to this figure, the tracker updating increases the performance. In another experiment, the synthetic data generation was removed from the tracking process. In this case, the trackers cannot follow the object of interest at all.

### 4.3. Visualization Result

**Figure 10:**
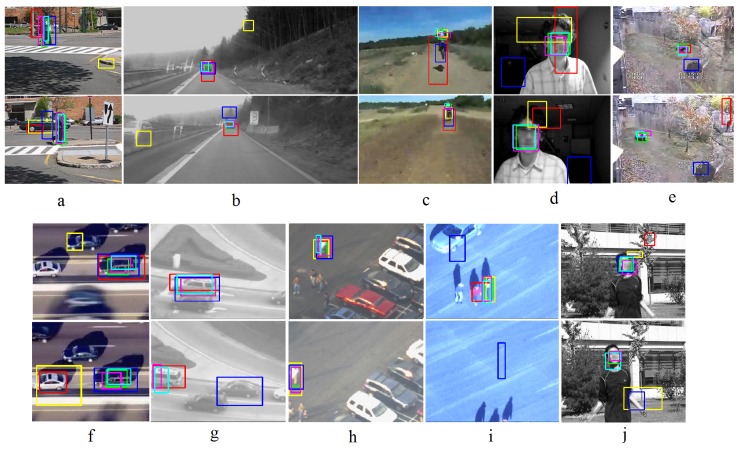
Visualization results of the trackers are shown and compared. The results of RCNN, FastRCNN, FasterRCNN, ACF, YOLO and Ground truth are shown with red, yellow, blue, magenta, cyan and green frames respectively. The figures from (**a**–**j**) show Pedestrain1, VW, Motocross, David, Panda, Carchase, Car, Pedestrain2, Pedestrain3 and Jump respectively. Each column includes two randomly selected frames of the same video.

The visualization comparison of the trackers for selected frames of the 10 videos and the ground truth is shown in Figure 10. From each video, two frames were randomly selected and shown in different rows. The sequence in Figure 10a (Pedestrain1) has similar and articular objects and pose change. The YOLO and ACF trackers can follow the pedestrian very well but the RCNN, FastRCNN and FasterRCNN trackers miss it. The video in Figure 10b (Volkswagen) is a long video which contains similar objects (cars), occlusion and illumination change. The RCNN, ACF and YOLO trackers show better results than FasterRCNN, and FastRCNN is tracking the background. In the first frame except for the FastRCNN tracker, the rest of the trackers can follow the car, but in the second frame only the YOLO and ACF trackers keep tracking the car. The YOLO tracker is more stable than ACF for this case. In this video, there are many frames without the desired car, but the RCNN and FastRCNN trackers track mistakenly the other car which is present in the scene. The sequence in Figure 10c (Motocross) includes appearance, illumination and scale change. In this example, except for RCNN, the rest of the trackers show good results. The video in Figure 10d (David) contains partial occlusion, scale and strong illumination change. In this case, RCNN, FastRCNN and FasterRCNN have poor results but the ACF and YOLO trackers show precise results. The sequence in Figure 10e (Panda) has occlusion, out-of-plane rotation, appearance and scale change. FastRCNN follows an incorrect object for the both shown frames and RCNN tracks one frame correctly and the other one wrongly. The others i.e., the YOLO, ACF and Fast RCNN trackers are working well. The video in Figure 10f (Carchase) is a long video which includes occlusion, similar objects, scale and illumination change. RCNN and Fast RCNN are mistakenly following another similar object whereas the FasterRCNN, YOLO and ACF-Trackers track the correct object. Figure 10g shows a sequence (Car) which has occlusion and similar objects. In this case, since the object (the white car) is leaving the scene, there is no ground truth for it. However, RCNN can completely and YOLO and ACF partially track the object, but, they are considered as a false positive. The FasterRCNN tracker tracks a similar object and the FastRCNN Tracker misses the object in both frames. The sequence in Figure 10h (Pedestrain3) includes similar objects and object occlusion. All the trackers except for YOLO can track the object of interest. In the first selected frame, YOLO detects partially the human but it misses the object of interest in the second frame. In this case, the YOLO human detection from the top view is poor. Figure 10i shows a video (Pedestrain2) which has occlusion and similar objects. For the first frame, the FastRCNN, YOLO and ACF trackers can track the object correctly but RCNN the tracker follows another human and FasterRCNN tracks a part of a car instead of the human. In the second frame, there is no human in the scene but, FasterRCNN tracks a point in the background. The video in Figure 10j (Jumping) contains strong movement and blurring. In this case, the ACF and YOLO-trackers track correctly. Generally, among the tested trackers, the ACF and YOLO trackers show better results.

### 4.4. Algorithm Speed Comparison

The trackers have been implemented on a hardware system with a specification as shown in Table 5. ACF, RCNN, FastRCNN and FasterRCNN were implemented using MATLAB. Except for the ACF tracker, the other trackers use GPU. The detection part the of YOLO tracker was done using C++ on GPU machine and the Kalman filter [54] was implemented using C++ on CPU.

**Figure 11:**
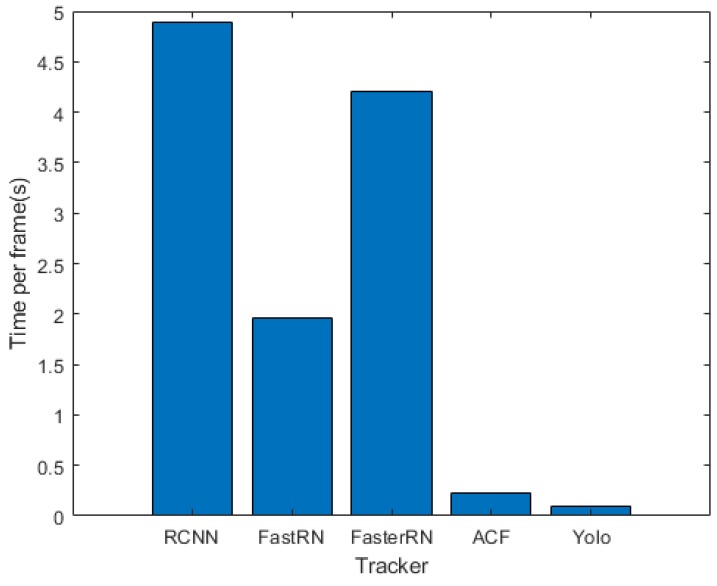
The trackers comparison in terms of the running time.

For each tracker, the average running time of all 10 videos has been measured and shown in Figure 11. The average fps(frame per second) for the RCNN, FastRCNN, FasterRCNN, ACF and YOLO trackers are 0.2, 0.5, 0.24, 4 and 9 respectively. As shown in Figure 11, the YOLO tracker has the fastest implementation because it does not use training while tracking. Among the online trackers, the ACF-tracker(implemented using CPU) shows has effective implementation than RCNN, FastRCNN and FasterRCNN (implemented using GPU). Thus, the ACF-tracker is faster than the rest of the online trackers. The main difference among the online trackers speed happens in the training phase and therefore ACF is even faster than FasterRCNN in this phase.

### 4.5. Discussion

The comparative study among the trackers is concluded as follows:

1. The ACF tracker has the best results among the online trackers from both accuracy and speed viewpoints. ACF has effective implementation, because it runs on the CPU machine instead of GPU for the other online trackers.
2. Among the RCNN-based trackers (i.e., RCNN, FastRCNN and FasterRCNN), RCNN has the best tracking accuracy. Though, FastRCNN and FasterRCNN are very fast in test phase, their tracking process is slow because they are very slow in training phase.
3. Since the YOLO tracker was implemented offline, it is the fastest tracker. YOLO is not qualified for online tracking, because it is very slow in training phase.
4. For human tracking from the front and side views, the combination of YOLO and Kalman filter shows the best results.
5. We recommend to use the ACF tracker in unknown objects tracking because YOLO does not detect them whereas the ACF tracker does.
6. Compared to YOLO and ACF, the RCNN-based trackers show less accuracy because they don’t have a very deep structure (i.e., 3 convolutional layers and 2 fully connected). By using a deeper convolutional neural network like YOLO the accuracy is dramatically increased.

The training vector length $T_{v}$ in Section 3 was investigated and showed that the online tracker follows the recent appearances of the object of interest. The length should be set to an optimal value, if it exceeds the value, the average accuracy decreases. On the other hand, selection of the shorter lengths leads to the under-fitting and low accuracy.

Some results of our research are not limited to tracking. They are stated as follows:

1. YOLO detector detects cars from the top view, but the object classification precision is low.
2. For human detection, since YOLO was biased to the data from the front view, although the YOLO detection results from the view is very good the classification results is disappointing.
3. Though, the object detection based on deep learning has recently improved, further improvement is still necessary. YOLO detection should be trained using a big dataset including more various views of the objects.

## 5. Conclusions

In this paper, we did a comprehensive comparative study in the context of object tracking using five famous recently proposed detectors. Two trackers based on online and offline tracking were used. The online tracker first generates positive and negative samples from the first frame and then trains detectors. The detector detects online the objects of interest in next frames and put them in a training vector. The detector is updated in certain intervals using the training vector. The detector detects the objects of interest in the next frames until the last frame. The ACF tracker showed the best results among the examined methods for the online tracking from both speed and accuracy perspectives. In the offline scenario, YOLO detector generates some candidates, then the tracker follows the object of interest using Kalman filter. Extensive experiments showed thatthe YOLO tracker outperforms the rest of the trackers. For the future work, YOLO detector will be trained using an updated dataset to improve the detection results from the top view. The experiments will be extended to other videos in VOT benchmark. We aim to extend our methods for multiple object tracking because all the detectors i.e., ACF, RCNN, FastRCNN, FasterRCNN and YOLO have the capability of multiple object detection. For the online trackers in each iteration of the training phase, instead of a single object, we will define multiple objects and the detectors will output different labels for different objects. In the case of offline tracking, YOLO is able to detect multiple objects as shown in Figure 5b. For each object, one Kalman filter will be used for tracking.

## Figures and Tables

**Table 1:** The number and types of the synthetic data are given in this table. The first row shows the total number of the synthetic data and each column shows the number and types of the synthetic data for a certain number of the synthetic data.

Total Number of the Synthetic Data	500	800	1000	2000	4000	10,000
First Frame	1	1	1	1	1	1
Additive Noise	2–9	2–9	2–9	2–9	2–9	2–9
Rotation	10–200	10–200	10–200	10–200	10–200	10–200
Rotation + Intensity Adjustment	201–400	201–599	201–400	201–400	201–400	201–400
Rotation + Contrast Enhancing	401–500	600–800	401–601	401–601	401–601	401–601
Brightness Change	-	-	602–1000	602–1000	602–1000	602–1000
Brightness Change + Resizing	-	-	-	1001–2000	1001–2000	1001–2000
Rotation + Brightness Change	-	-	-	-	2001–4000	2001–4000
Last Row + Intensity Adjustment	-	-	-	-	-	4001–10,000

**Table 2:** Parameter sets for the online trackers.

	$T_{1}$	$T_{2}$	$T_{3}$	$T_{4}$	$T_{5}$	$T_{6}$	$S_{1}$	$S_{2}$	$S_{3}$	$S_{4}$	$S_{5}$	$S_{6}$	$S_{7}$
SET1	200	300	500	1000	2000	5000	10	20	50	100	500	1000	-
SET2	200	410	450	500	1000	2000	20	30	40	50	100	500	1000

**Table 3:** Parameter set 1 regarding the online trackers.

Methods	Length of $T_{v}$	Length of Synthetic Data	Stage	Epoch
ACF1	614	210	5	-
ACF2	1000	500	3	-
RCNN1, FastRCNN and FasterRCNN	1000	500	-	3
RCNN2	4000	4000	-	10

**Table 4:** Comparison of the trackers in terms of Recall (left number), Precision (left number) and F-measure (right number), the abbreviations of the videos are $p_{i}$: pedestrian i, Moto: motocross, VW:Volkswagen and CarC:Carchase.

Method	David	Jump	P1	P2	P3	Car	Moto	VW	CarC	Panda
ACF1	1/0.98/0.99	0.96/1/0.98	0.96/0.88/0.92	0.90/1/0.95	0.94/0.85/0.89	0.97/0.95/0.96	0.87/0.35/0.50	0.14/0.13/0.13	0.4/0.74/0.52	0.83/0.69/0.75
ACF2	0.99/0.99/0.99	0.87/0.99/0.93	0.96/0.82/0.88	0.84/1/0.91	0.93/0.96/0.94	0.93/0.98/0.95	0.97/0.56/0.71	0.78/0.65/0.71	0.78/0.64/0.70	0.84/0.85/0.85
RCNN1	0.69/0.27/0.39	0.96/0.22/0.36	0.95/0.15/0.26	0.61/0.25/0.35	0.99/0.69/0.81	0.97/0.35/0.51	1/0.42/0.59	1/0.35/0.52	0.95/0.37/0.53	0.99/0.11/0.20
RCNN2	0.97/0.48/0.64	0.96/0.36/0.52	0.74/0.15/0.25	0.75/0.56/0.64	0.99/0.67/0.80	0.99/0.90/0.94	0.98/0.40/0.57	0.96/0.57/0.72	0.94/0.45/0.61	0.99/0.37/0.54
FastRCNN	0.90/0.19/0.31	1/0.01/0.02	1/0.29/0.45	0.80/0.21/0.33	1/0.73/0.84	0/0/0	1/0.31/0.47	1/0.13/0.23	0.99/0.29/0.45	0.40/0.07/0.12
FasterRCNN	0.36/0.17/0.23	0.95/0.41/0.57	1/0.33/0.5	0.90/0.81/0.85	1/0.62/0.77	1/0.86/0.92	0.95/0.16/0.27	0.75/0.09/0.16	0.92/0.52/0.66	1/0.07/0.13
Offline ACF	0.72/0.49/0.58	0.30/1/0.46	0.40/0.49/0.44	0.20/1/0.33	0.89/0.99/0.93	0.59/0.81/0.68	1/0.39/0.56	0.43/0.66/0.52	0.45/0.68/0.54	0.08/0.83/0.15
YOLO	1/0.99/0.99	1/1/1	1/0.77/0.87	1/0.43/0.60	1/0.59/0.74	0.99/0.0.93/0.96	0.89/0.82/0.85	0.99/0.86/0.92	0.97/0.92/0.94	0.99/0.78/0.88

**Table 5:** The specification of the hardware system for our experiments.

	Brand	Specification
CPU	Intel	Core i7-7700K CPU @ 4.20 GHz
GPU	GeForce	GTX 1080 Ti/PCIe/SSE2
Ram	Kingston	15.6 GB
